# Uncovering the cellular and omics characteristics of natural killer cells in the bone marrow microenvironment of patients with acute myeloid leukemia

**DOI:** 10.1186/s12935-024-03300-w

**Published:** 2024-03-14

**Authors:** Leisheng Zhang, Yunyan Sun, Chun-e Xue, Shuling Wang, Xianghong Xu, Chengyun Zheng, Cunrong Chen, Dexiao Kong

**Affiliations:** 1 Science and Technology Innovation Center, The Fourth People’s Hospital of Jinan, The Teaching Hospital of Shandong First Medical University, 50 Shifan Road, Tianqiao District, Jinan, 250031 Shandong China; 2 National Health Commission (NHC) Key Laboratory of Diagnosis and Therapy of Gastrointestinal Tumor, Gansu Provincial Hospital, Lanzhou, 730000 China; 3 Department of Hematology, Peking University Cancer Hospital Yunnan, Yunnan Cancer Hospital, The Third Affiliated Hospital of Kunming Medical University, Kunming, 650118 China; 4 Department of Hematology, Langfang City Hospital of Traditional Chinese Medicine, Langfang, 065000 China; 5 Department of Critical Care Medicine, Fujian Medical University Union Hospital, Fuzhou, 350001 China; 6 Department of Hematology, The Second Hospital, Cheeloo College of Medicine, Shandong University, Jinan, 250033 China

**Keywords:** Natural killer (NK) cells, Acute myeloid leukemia (AML), Bone marrow microenvironment, Cellular vitality, Omics analysis

## Abstract

**Background:**

Acute myeloid leukemia (AML) is a highly heterogeneous hematologic malignancy and the most frequently acute leukemia of stem cell precursors and the myeloid derivatives in adult. Longitudinal studies have indicated the therapeutic landscape and drug resistance for patients with AML are still intractable, which largely attribute to the deficiency of detailed information upon the pathogenesis.

**Methods:**

In this study, we compared the cellular phenotype of resident NK cells (rAML-NKs, rHD-NKs) and expanded NK cells (eAML-NKs, eHD-NKs) from bone marrow of AML patients (AML) and healthy donors (HD). Then, we took advantage of the co-culture strategy for the evaluation of the in vitro cytotoxicity of NK cells upon diverse tumor cell lines (e.g., K562, Nalm6, U937). With the aid of RNA-sequencing (RNA-SEQ) and bioinformatics analyses (e.g., GOBP analysis, KEGG analysis, GSEA, volcano plot), we verified the similarities and differences of the omics features between eAML-NKs and eHD-NKs.

**Results:**

Herein, we verified the sharp decline in the content of total resident NK cells (CD3^−^CD56^+^) in rAML-NKs compared to rHD-NKs. Differ from the expanded eHD-NKs, eAML-NKs revealed decline in diverse NK cell subsets (NKG2D^+^, CD25^+^, NKp44^+^, NKp46^+^) and alterations in cellular vitality but conservations in cytotoxicity. According to transcriptomic analysis, AML-NKs and HD-NKs showed multifaceted distinctions in gene expression profiling and genetic variations.

**Conclusions:**

Collectively, our data revealed the variations in the cytobiological and transcriptomic features between AML-NKs and HD-NKs in bone marrow environment. Our findings would benefit the further development of novel biomarkers for AML diagnosis and NK cell-based cytotherapy in future.

**Supplementary Information:**

The online version contains supplementary material available at 10.1186/s12935-024-03300-w.

## Background

Acute myeloid leukemia (AML) has been recognized as a heterogeneous hematological malignancy and the most common acute leukemia in adults, which is mainly caused by multifaceted somatic mutations in myeloid differentiation and monoclonal proliferation of immature progenitors [1, 2]. Despite the approval and application of novel drugs for patients, AML still remains as a major field of unmet medical need among diverse hematologic malignancies attributes to the deficiency of details information upon the cytopathologic and pathogenic features [3]. For decades, considerable progresses have been achieved in elucidating the pathogenesis of AML both at the cellular and molecular levels [4]. For instance, diverse inherited genetic loci and novel biomarkers (e.g., epigenetic, genetic, and protein) involved in myelodysplastic syndrome (MDS) and AML development in patients have been identified for the risk stratification and treatment assessment of AML patients on the basis of epigenetic and omics profiles [3, 5]. According to the World Health Organization (WHO) Classification of leukemia and myeloid neoplasms, AML can be divided into eight categories according to the recurrent genetic abnormalities and into three categories according to the indicated gene mutations. However, the genes with sufficient accuracy for unraveling the risk-stratification schemes and clinically targetable treatment decision-making of AML are still largely obscure [6].

Natural killer (NK) cells are critical innate lymphoid cells (ILCs) for their effect in tumor immunosurveillance and antiviral immunity dispense with presensitization, which have been involved in both innate and adoptive immune responses via the cytotoxic and cytokine-secreting approaches [7–9]. NK cells have been considered as heterogeneous populations generated from hematopoietic stem cells (HSCs) in the bone marrow environment (BME) with limited functional and phenotypic diversity, which are composed of two distinct subsets in human, including the cytotoxic CD3^−^CD56^dim^CD16^high^ and the IFN-γ-producing CD3^−^CD56^bright^CD16^+^ counterparts [10, 11]. To date, adoptive NK cells and the concomitant chimeric antigen receptor-transduced NK cells (CAR-NKs) have been explored in various cancer settings including AML, and aiming to attain better therapeutic outcomes [12, 13]. For example, Terrén et al. and Albinger et al. reported the application of cytokine-induced memory-like (CIML) NK cells and primary CD33-targeting CAR-NK cells for the treatment of patients with AML, respectively [14, 15]. Furthermore, Soldierer et al. and Xu et al. highlighted the genetic engineering of human cells for the CAR-enhanced immunotherapy of hematological malignancies including AML [16, 17]. Very recently, Gauthier and the colleagues reported the successful control of AML by a trifunctional NKp46^−^CD16a^−^NK cell engager (NKCE) with prolonged pharmacodynamic effects and very low inflammatory cytokine induction against CD123 antibody-dependent cell cytotoxicity (ADCC) [18]. Of note, Crinier et al. showed the trajectories of NK cell differentiation from the resident CD56^bright^ NK cells to the CD56^dim^ NK1-like NK cells and the CD56^bright^ NK2-like NK cells, yet the major cytophenotypic and omics features of resident and expanded NK cells in the bone marrow of AML patients (AML-NKs) are largely obscure.

For the purpose, in this study, we isolated resident NK cells (rNKs) from both AML patients (rAML-AMLs) and healthy donors (rHD-AMLs), and conducted ex vivo NK cell expansion and activation from the corresponding rNKs after a 14-day’s induction (eAML-AMLs, eHD-AMLs). With the aid of multifaceted cellular and molecular analyses, we verified the cytophernotic (e.g., cytomorphology, immunophenotyping, cellular vitality, and cytotoxicity against diverse tumor cell lines) and transcriptomic characteristics (e.g., gene expression profiling, genetic variations) between AML-NKs and HD-NKs in the bone marrow microenvironment, which collectively indicated the pathogenic effect of NK cells in patients with AML and would benefit the further development of novel biomarkers for clinical diagnosis and NK cell-based cytotherapy in future.

## Methods

### Preparation of mononuclear cells (MNCs) from bone marrow

Human bone marrow was obtained with the consent of AML patients and healthy donors (HD) and the approval of the Ethics Committee of Gansu Provincial Hospital and the guideline of Helsinki Declaration (2023-120). For MNCs isolation from bone marrow of AML patients (AML-MNCs) and HD (HD-MNCs), the Ficoll-based density gradient centrifugation were conducted as we described recently with several modifications [19, 20]. As sample collection, 3–5 ml fresh bone marrow in 10 HD (healthy donors satisfied the selection criteria for bone-marrow transplantation donation) and 9 AML patients before treatment (without receiving clinical treatment course and regimen) was collected by bone marrow aspiration in sterile anti-coagulant tubes with inverted mixing for 10 times. Then, the blood samples were immediately turned to MNC isolation as abovementioned according to the manufacturer’s instructions. The detail data of AML was available in Additional file 1: Table S1.

### NK cell expansion and activation from MNCs

For ex vivo NK cell expansion and activation, 2 × 10^6^/ml HD-MNCs or AML-MNCs were seeded in NK MACS basal Medium (Miltenyi Biotech, Germany) with the indicated recombinant human interleukin (rhIL) addition, including 1000U/ml rhIL-2, 10 ng/ml rhIL-15, and 50 ng/ml rhIL-18 [20]. The medium was half replaced every other day for 7 days, and then completely replaced every day for the other 7 days as we previously reported [19, 21]. The numbers of total NK cells (CD3^−^CD56^+^) were calculated based on Trypan Blue-based viable cell counting, and the proportions of total NK cells and the subsets (CD16^+^, NKG2A^+^, NKG2D^+^, NKp44^+^, NKp46^+^) were detected by flow cytometry (FCM) analysis at day 0 and day 14 of ex vivo NK cell induction. The list of the aforementioned cytokines was shown in Additional file 1: Table S2.

### Flow cytometry (FCM) analysis

FCM analysis was performed as we recently reported [20, 22]. In brief, the fresh-enriched MNCs (day 0) and the MNC-derived cells (day 14) were harvested by centrifugation at 300×*g* for 5 min and resuspended by 1 × PBS (Solarbio, China) for twice. After that, the cells were incubated in 1 × PBS (Solarbio, China) with 2% fetal bovine serum (FBS) (Australia) and the fluorescence-conjugated antibodies such as anti-CD3-PE (BioLegend, USA), anti-CD3-APC (BioLegend, USA), anti-CD4-PE (BioLegend, USA), anti-CD8-PE-Cy7 (BioLegend, USA), anti-CD56-APC (BioLegend, USA), anti-CD16-FITC (BioLegend, USA), anti-CD25-FITC (BioLegend, USA), anti-NKG2D-perCP-Cy5.5 (BioLegend, USA), anti-NKp44-APC-Cy7, anti-NKp46-PE-Cy7 (BD Biosci, USA), anti-NKG2A-PE (BD Biosci, USA), anti-CD107a-PE (BD Biosci, USA), 7-AAD (BD Pharmigen), Propidium iodide (PI) (BD Pharmigen, USA) or Annexin V-FITC (Tianjin Sungene Biotech, China) in dark for 30 min. Finally, the cells were washed and turned to FACS Canto II (BD Biosci, USA) and FlowJo 10.0 software (Tree Star, USA) for analysis. The list of the indicated antibodies was available in Additional file 1: Table S3.

### Cell cycle assessment of eHD-NKs and eAML-NKs

Cell cycle assessment of the MNCs (HD-MNCs, AML-MNCs) and the derivatives at the indicated time points was conducted as we reported [19, 23]. In brief, the cells were harvested and suspended in 1 × PBS (Solarbio, China), and then incubated with anti-CD3-PE and anti-CD56-APC antibodies for 20 min in dark. After that, the cells were incubated in 70% (v/v) ethanol (Thermo Fisher Scientific, USA) and fixed for 24 h, and followed by centrifugation at 1000×*g* for 5 min and resuspended with 1 × PBS (Solarbio, China) at 4 ℃ for twice. Then, the cells were incubated with PI staining solution (BD Pharmigen, USA) for 30 min at 37 ℃ and detected by BD LSR II (BD Biosci, USA) and the ModFit software (Verity Software House Co. Ltd, USA).

### Cell apoptosis analysis of eHD-NKs and eAML-NKs

The proportions of apoptotic eHD-NKs and eAML-NKs were assessed with the Annexin V Apoptosis Detection Kit (Tianjin Sungene Biotech, China) according to the manufactures’ instructions as we reported before [19, 24]. In details, 1 × 10^6^ cells at day 14 (eHD-NKs, eAML-NKs) were harvested and resuspended in precooled 1 × PBS (Solarbio, China). Then, the cells were respectively incubated in 100 μl 1 × Binding Buffer, Annexin V-FITC and 7-AAD solution (Tianjin Sungene Biotech, China) in dark. Finally, the proportions of apoptotic cells in eHD-NKs and eAML-NKs were detected by FACS Canto II (BD Biosci, USA) and analyzed by FlowJo 10.0 software (Tree Star, USA).

### Cytotoxicity assessment of NK cells

The cytotoxicity of eHD-NKs and eAML-NKs was evaluated as we recently reported [20, 24]. For preparation, the indicated tumor cell lines (K562, human myeloid leukemia cell line; Nalm6, human lymphocytic leukemia cell line; U937, human myeloid leukemia cell line) were co-cultured in RPMI-1640 medium (Gibco, USA) supplemented with 10% FBS (Gibco, USA) at 37 ℃, 5% CO_2_ as we recently described [20, 25]. In brief, the indicated tumor cell lines were incubated with CellTrace Violet reagent (BV421 laser channel, Invitrogen, USA). Then, the tumor cells were solely (negative control, NC) or co-cultured with eHD-NKs or eAML-NKs at the effector-to-target ratio (E: T = 1: 3) for 8 h. After that, the cells were incubated with the indicated antibodies (anti-CD3-FITC, ant-CD56-APC, anti-CD-107-PE) (BioLegend, USA) and 7-AAD solution (Tianjin Sungene Biotech, China). Then, the cells were resuspended in 200 µl 1 × PBS (Solarbio, China) and 5 µl Precision Count Beads (BioLegend, USA). Finally, the percentage of the cells were detected by FACS Canto II (BD Biosci, USA) and analyzed by FlowJo 7.0 software (Tree Star, USA) as we described before [19, 21]. Cytotoxicity of eHD-NKs or eAML-NKs was calculated according to the formula: Cytotoxicity = (1 − N_1_/N_0_) × 100%. N_0_ and N_1_ represent the total number of living K562 cells in the negative control group and the experimental group, respectively [19, 20].

### RNA-sequencing (RNA-SEQ) and bioinformatics analysis

Total mRNAs were harvested from the indicated eHD-NKs and eAML-NKs by utilizing the TRIZol reagent (ThermoFisher, USA) according to the manufactures’ instructions as we recently reported [19, 26, 27]. For RNA-SEQ analysis, the enriched mRNAs were turned to quality test and sent to BGI Genomics (Shenzhen, China) for sequencing. Multidimensional bioinformatics analyses of the RNA-SEQ data were accomplished with the online database and platforms, including Gene Set Enrichment Analysis (GSEA), Volcano Plot, HeatMap, Venn diagram, Principal Component Analysis (PCA), Gene Ontology (GO), and Kyoto Encyclopedia of Genes and Genomes (KEGG) as we previously described [22, 27, 28].

### Statistical analysis

All data were analyzed with the aid of the GraphPad Prism 8.0 software (GraphPad Software Inc., USA) as we recently described [24, 28]. In brief, the data of two unpaired groups were turned to the Student's unpaired t test was utilized for analysis, whereas the data of multiple unpaired groups were analyzed by utilizing the one-way ANOVA method, respectively. All data were shown as mean ± standard error of mean (SEM) (N = 3 independent experiments). Significant statistical difference was considered only when P value was less than 0.05 (P < 0.05). *, P < 0.05; **, P < 0.001; ***, P < 0.001; ****, P < 0.0001; NS, not significant.

## Results

### The bone marrow of AML patients revealed sharp decline in resident NK cells and variations in the subsets

**Fig. 1:**
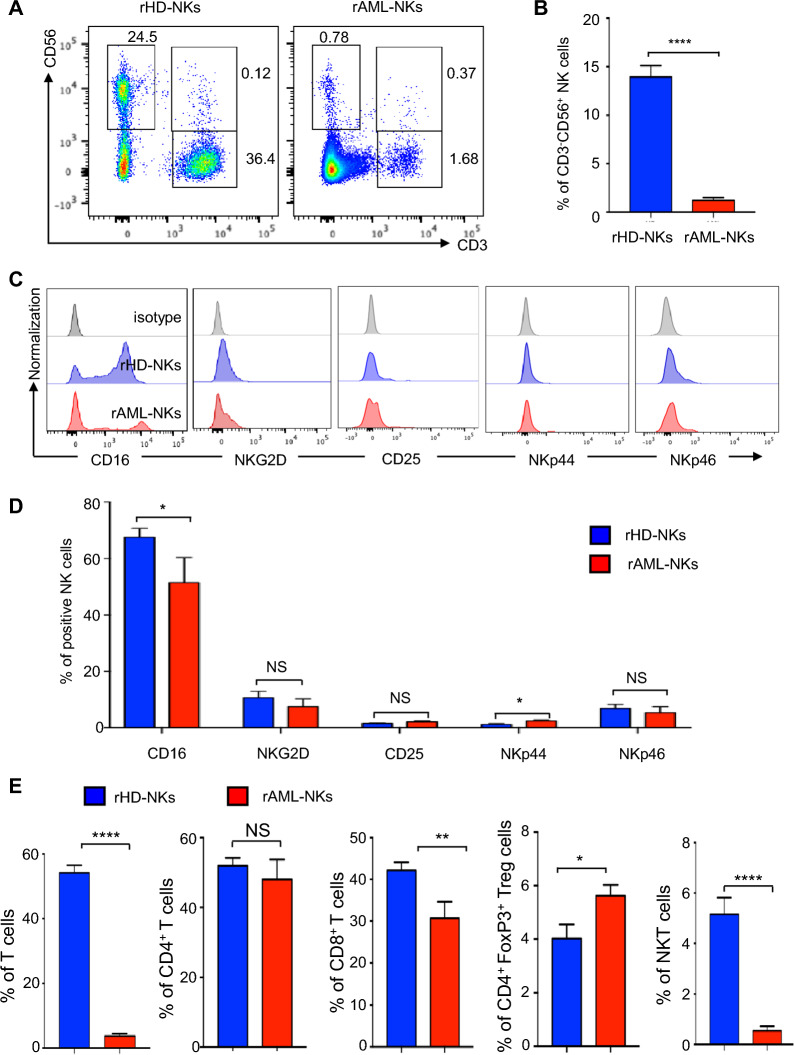
Comparison of the phenotype and components of rHD-NKs and rAML-NKs. **A**, **B** Representative FCM diagrams (**A**) and statistical analysis (**B**) of total (CD3^−^CD56^+^) NK cells in the rHD-NKs and rAML-NKs groups. **C**, **D** Representative FCM diagrams (**C**) and statistical analysis (**D**) of total activated (CD3^−^CD56^+^CD16^+^) NK cells and the subsets (NKG2D^+^, CD25^+^, NKp44^+^, NKp46^+^) in the rHD-NKs and rAML-NKs groups. **E** Statistical analysis of total CD3^+^CD56^−^ T lymphocytes, CD3^+^CD4^+^ T cells, CD3^+^CD8^+^ T cells, CD4^+^FoxP3^+^ T cells and CD3^+^CD56^+^ NKT cells in the rHD-NKs and rAML-NKs groups. All data were shown as mean ± SEM (N = 3 independent experiments). *P < 0.05; **P < 0.01; ****P < 0.0001; NS: not significant

To verify the potential variations of NK cells between AML patients and HDs, we isolated MNCs from bone marrow and found that the percentage of total resident AML-NK cells (rAML-NKs) was sharply declined when compared to rHD-NKs (Fig. 1A, B). Furthermore, the proportion of total activated CD3^−^CD56^+^ NKs in rAML-NKs was also lower than that in the rHD-NKs group, whereas the NKp44^+^ subset revealed reverse tendency (Fig. 1C, D). Instead, minimal differences were observed in the content of relative NK cell subpopulations between rHD-NKs and rAML-NKs, including the activated CD25^+^, and NKG2D^+^ and NKp46^+^ subsets (Fig. 1C, D, Additional file 2: Fig. S1A, S1B). Additionally, AML-MNCs showed sharp decline in the proportion of total T cells (CD3^+^) and CD3^+^CD56^+^ NKT cells (Fig. 1E, Additional file 2: Fig. S1C). As to the indicated subsets of T cells, we noticed the diverse variations in contents in AML-MNCs compared to HD-MNCs, including the moderate decrease in CD3^+^CD8^+^ T cells, the moderate increase in CD4^+^FoxP3^+^ Treg cells, and the minimal differences in CD3^+^CD4^+^ T cells (Fig. 1E). Taken together, our data indicated the variations in the content of total rAM-NKs in bone marrow environment.

### The expanded AML-NKs showed deficiency in ex vivo amplification and variations in subpopulations

**Fig. 2:**
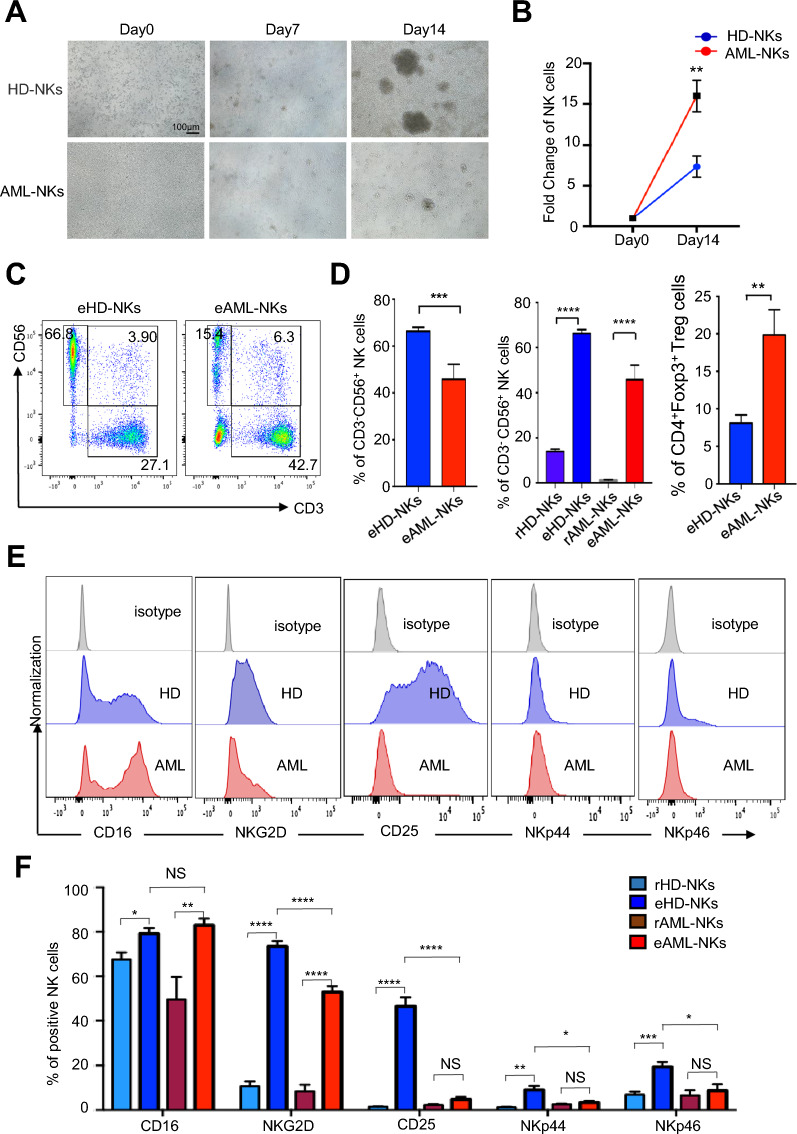
Comparison of the phenotype and components of eHD-NKs and eAML-NKs. **A** Phase contrast images of HD-NKs and AML-NKs at the indicated time points (day 0, 7, 14) during the 14-days’ in vitro amplification and activation. Scale bar = 100 μm. **B** Fold change of NK cells in the HD-NKs and AML-NKs groups. **C**, **D** Representative FCM diagrams of total (CD3^−^CD56^+^) NK cells in expanded NK cells (eHD-NKs, eAML-NKs) (**C**) and statistical analysis (**D**) of total (CD3^−^CD56^+^) NK cells in resident NK cells (rHD-NKs, rAML-NKs) and expanded NK cells (eHD-NKs, eAML-NKs). **E**, **F** FCM diagrams (**E**) and statistical analysis (**F**) of total activated NK cells (CD3^−^CD56^+^CD16^+^) and the subsets (NKG2D^+^, CD25^+^, NKp44^+^, NKp46^+^) in the indicated groups. All data were shown as mean ± SEM (N = 3 independent experiments). *P < 0.05; **P < 0.01; ***P < 0.001; ****P < 0.0001; NS: not significant

To further dissect the cytophenotypic characteristics of HD-NKs and AML-NKs, we turned to our well-established “3ILs”-based strategy for ex vivo NK cell amplification and activation from HD-MNCs and AML-MNCs [19, 29]. Intuitively, we observed the decrease in cell aggregates and unicellular number in the AML-NKs group compared to the HD-NKs group at day 14 of the NK cell induction (Fig. 2A, B). With the aid of FCM analysis, we found that the percentage of total CD3^−^CD56^+^ NK cells in the expanded NK cells (eHD-NKs, eAML-NKs) were promoted substantially improved compared to the corresponding resident NK cells (rHD-NKs, rAML-NKs), whereas the eAML-NKs group showed moderate decline in total NK cells compared to the eHD-NKs group (Fig. 2C, D).

Furthermore, we found the percentage of total activated CD3^−^CD56^+^ NK cells in the eAML-NKs group was equal to that in the eHD-NK group (Fig. 2E, F). As to the subsets of expanded NK cells, we noticed the significant decrease in the proportions of diverse subpopulations (NKG2D^+^, CD25^+^, NKp44^+^, NKp46^+^) in the eAML-NKs group, and in particular, the CD25^+^ cells typically associated with NK cell activation and cytotoxic function revealed sharp decline in eAML-NKs compared to eHD-NKs (Fig. 2E, F, Additional file 3: Fig. S2A). Collectively, these data showed the multidimensional deficiency in ex vivo amplification and activation of eAML-NKs compared to eHD-NKs.

### eAML-NKs manifested diversity in cellular vitality and similarity in vitro cytotoxicity with eHD-NKs

**Fig. 3:**
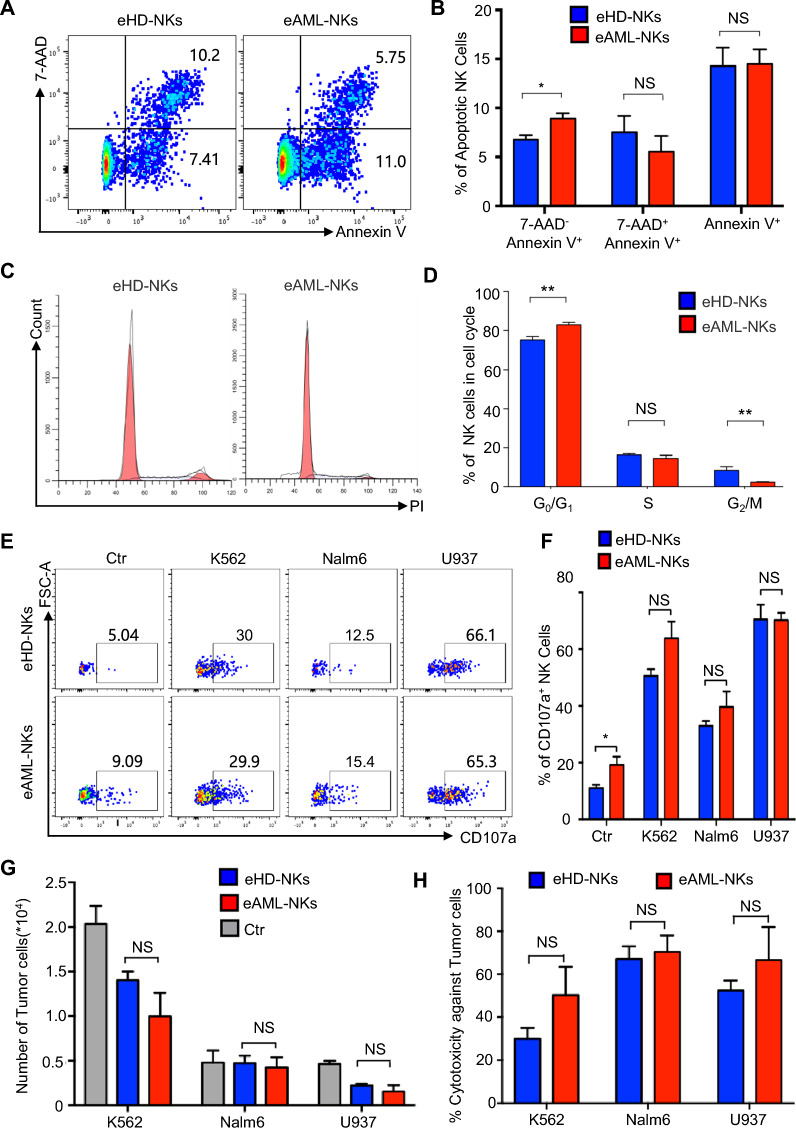
Comparison of the cellular vitality and cytotoxicity of eHD-NKs and eAML-NKs upon tumor cell lines. **A**, **B** FCM diagrams (**A**) and statistical analysis (**B**) of the apoptotic cells (7-AAD^−^Annexin V^+^, 7-AAD^+^Annexin V^+^, Annexin V^+^) in eHD-NKs and eAML-NKs. **C**, **D** FCM diagrams (**C**) and statistical analyses (**D**) of the distribution of the indicated sub-stages (G0/G1, S, G2/M) in cell cycle. **E**, **F** FCM diagrams (**E**) and statistical analysis (**F**) of CD107a^+^ NK cells in the Ctr group (monoculture) and the experimental groups cocultured with diverse tumor cell lines (K562, Nalm6, U937) at the effector-to-target ratio (E: T = 1:3). **G**, **H** Comparison of the ex vivo cytotoxicity of eHD-NKs and eAML-NKs against the indicated tumor cell lines (K562, Nalm6, U937) (E: T = 1:3). All data were shown as mean ± SEM (N = 3 independent experiments). All data were shown as mean ± SEM (N = 3 independent experiments). *P < 0.05; **P < 0.01; NS: not significant

Having verified the cellular phenotypes between HD-NKs and AML-NKs, we are aiming to verify the cellular vitality of the indicated NK cells. As shown by Fig. 3A, B, the percentage of 7-AAD^−^ Annexin V^+^ apoptotic eAML-NKs was increased compared to that in eHD-NKs, whereas minimal differences were observed in the 7-AAD^+^Annexin V^+^ and Annexin V^+^ subsets (also see Additional file 3: Fig. S2B). According to the distributions of sub-stages of cell cycle, a higher percentage of CD3^−^CD56^+^ eAML-NKs stayed in G0/G1 stage, whereas a lower percentage of the cells located in the G2/M stage when compared with the eHD-NKs group (Fig. 3C, D).

To further explore the in vitro cytotoxic activity of eAML-NKs and eHD-NKs, we turned to ex vivo tumor-killing model by coculturing the aforementioned eNK cells with diverse targeted tumor cell lines, including K562 cells (human chronic myeloid leukemia cell line), Nalm6 cells (human B lympholeukemia cell line), and U937 cells (human histiocytic lymphoma cells). As shown by the FCM diagrams and statistical analysis, the percentages of eAML-NKs with CD107a expression at the effector-to-target ratio (E: T = 1:3) was comparable to those in the eHD-NKs groups (Fig. 3E, F). Meanwhile, we consistently observed the minimal differences between eAML-NKs and eHD-NKs in killing capacity against the indicated tumor cell lines based on the calculation of the residual living tumor cells (Fig. 3G, H). Taken together, these data indicated the diverse variations in cellular vitality and conservations in cytotoxicity between eAML-NKs and eHD-NKs.

### The multifaceted variations in the landscape of gene expression profile between HD-NKs and AML-NKs

**Fig. 4:**
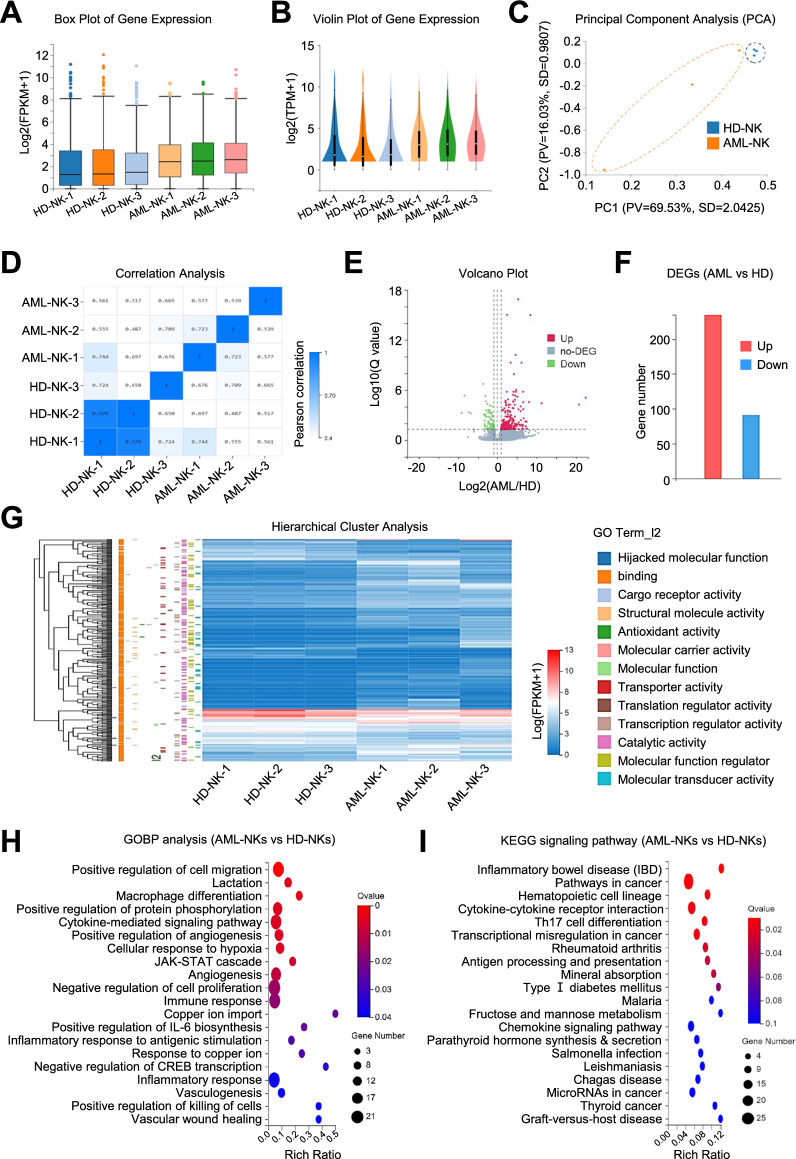
The landscape of gene expression pattern of eHD-NKs and eAML-NKs. **A**, **B** Box plot (**A**) and Violin plot (**B**) showed the distribution of genes expression pattern in eHD-NKs (HD-NK-1, HD-NK-2, HD-NK-3) and eAML-NKs (AML-NK-1, AML-NK-2, AML-NK-3). **C**, **D** Principal component analysis (PCA) and correlation analysis (**D**) of the aforementioned NK cells (eHD-NKs, eAML-NKs). **E**, **F** Volcano plot diagram (**E**) and bar chart (**F**) revealed the up-regulated (Up) and downregulated (Down) DEGs, together with the non DEGs (no-DEG) between eHD-NKs and eAML-NKs. **G** HeatMap diagram showed the hierarchical cluster pattern and GO terms of DEGs between the indicated groups (eHD-NKs, eAML-NKs). **H**, **I** Gene Ontology Biological Process (GOBP) analysis (**H**) and KEGG signaling pathway analysis (**I**) of the DEGs between eHD-NKs and eAML-NKs

Having clarified the cytophenotypic characteristics, we next turned to dissect the similarities and differences between HD-NKs and AML-NKs at omics features. For the purpose, we took advantage of RNA-SEQ and multidimensional bioinformatics analyses, and found that HD-NKs and AML-NKs revealed variations in general gene expression pattern according to the box plot and violin plot of gene expression (Fig. 4A, B). With the aid of principal component analysis (PCA), we intuitively observed the distant genetic affiliation between the indicated groups, which was further confirmed by Pearson correlation analysis (Fig. 4C, D).

Simultaneously, a cohort of upregulated (Up) and downregulated (Down) differentially expressed genes (DEGs) were enriched according to the log_10_ (Q value) of the indicated genes in HD-NKs and AML-NKs (Fig. 4E, F). In details, as shown by the hierarchical cluster analysis of HeatMap diagram, the DEGs were enriched in diverse gene ontology (GO) terms such as cargo receptor activity, translation regulator activity and molecular function regulator (Fig. 4G). Furthermore, by conducting GOBP analysis, we found the aforementioned DEGs were involved in biological processes, including positive regulation of cell migration, negative regulation of cell proliferation, cytokine-mediated signaling pathway, JAK-STAT cascade and inflammatory response (Fig. 4H). According to KEGG analysis, the DEGs between HD-NKs and AML-NKs mainly participated in pathways in cancer, cytokine-cytokine receptor interaction, immune response (e.g., Th17 cell differentiation, antigen processing and presentation, chemokine signaling pathway) (Fig. 4I). Taken together, AML-NKs revealed multifaceted variations with HD-NKs in gene expression profiling and especially the DEGs related with cellular vitality- and immune response-associated biological processes.

### The variations in enriched gene sets and the spectrum of the genetic spectrum between HD-NKs and AML-NKs

**Fig. 5:**
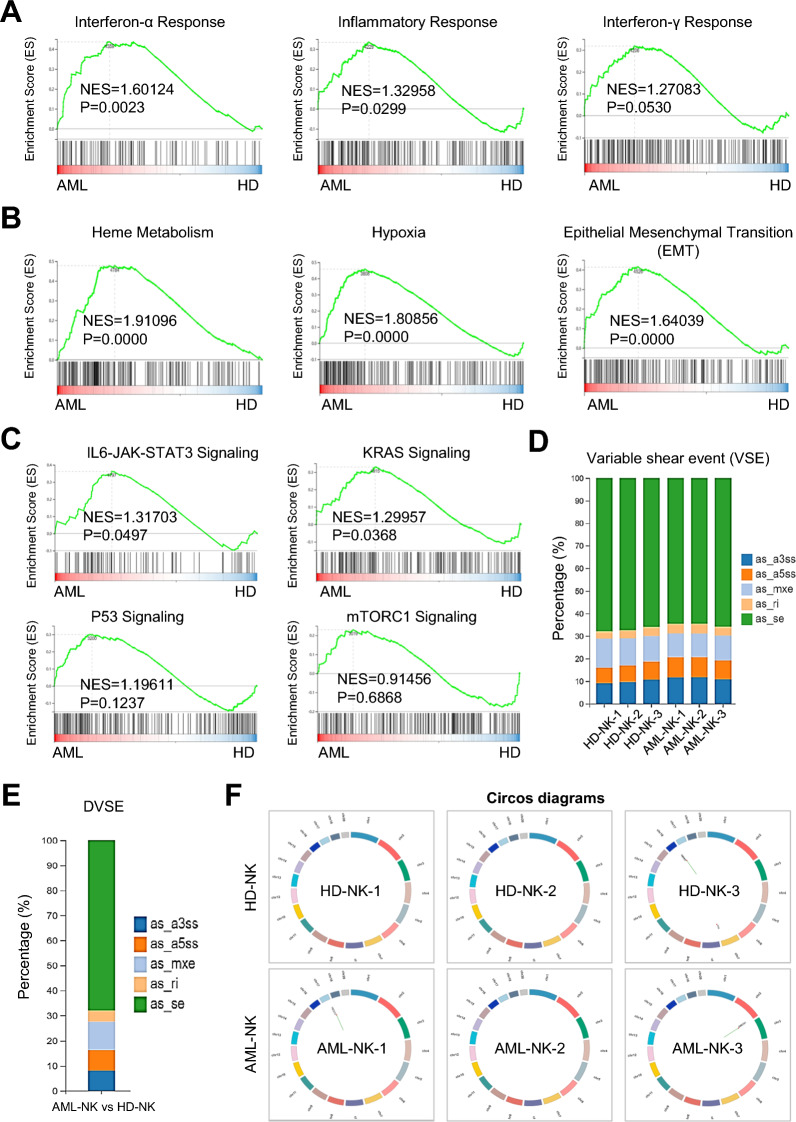
Comparison of the gene sets and somatic variations between eHD-NKs and eAML-NKs. **A**–**C** Gene set enrichment analysis (GSEA) sowed the differentially expressed gene sets, including those involved in representative immune response (**A**), biological processes (**B**), and signaling pathways (**C**). **D**, **E** The distribution of genes with somatic variations, including the variable shear events (VSE) (**D**) and the differentially VSEs (DVSE) (**E**). **F** Circos diagrams revealed the distribution of genes with the indicated somatic variations in the chromosomes (SNPs, INDELs, and gene fusion events)

To further illuminate the omics features of HD-NKs and AML-NKs, we turned to gene set enrichment analysis (GSEA) and noticed the specific enrichment of the significantly different gene sets associated with interferon-α response (P < 0.0023) and inflammatory response (P = 0.0299) rather than inflammatory response (P = 0.0530) (Fig. 5A). Meanwhile, gene sets involved in multiple biofunctions were also enriched, including heme metabolism, hypoxia and epithelial mesenchymal transition (EMT) (P < 0.0001) (Fig. 5B). As to those with signaling pathways, we observed the gene sets with significant differences between HD-NKs and AML-NKs were mainly related with IL6-JAK-STAT3 signaling and KRAS signaling rather than the P53 signaling or m TORC1 signaling instead (Fig. 5C).

Subsequently, we compared the spectrum of genes with genetic modifications, we meticulously dissected the overview of the indicated variable shear events (VSE), and verified the conservations in the distribution of as_se, as_ri, as_mxe, as_a3ss, and as_a5ss between HD-NKs and AML-NKs (Fig. 5D). Of them, the as_se subset is the dominant differentially VSEs (DVSE) between HD-NKs and AML-NKs (Fig. 5E). In addition, according to the Circos diagrams, there were minimal differences between HD-NKs and AML-NKs in the loci regional distribution of the aforementioned somatic variations in the chromosomes, including SNPs, INDELs, and gene fusion events (Fig. 5F). Collectively, these data indicated the variations in diverse gene sets and conservations in genetic spectrum between HD-NKs and AML-NKs.

## Discussion

As the most common malignant disease of myeloid precursors in adult, AML with various genetic abnormalities has caused great difficulty for accurate risk stratification and treatment intensity [30]. Despite the progress of novel agents for the treatment paradigm, yet the optimal regimens for AML patients remain absent due to the limited guidance and the deficiency of pathogenesis [31]. For the purpose, in this study, we prepared resident and expanded AML-NKs and HD-NKs, and verified the alterations in the content of total NK cells and the subpopulations, together with the cellular vitality and cytotoxic activity. Furthermore, by conducting omics analysis, we illuminated the gene expression spectrum and somatic variations between AML-NKs and HD-NKs. Collectively, our data would collectively supply new references for further dissecting the pathogenesis and clinical diagnosis of AML from the viewport of NK cells in bone marrow environment.

To overcome the poor prognosis of AML, we and other investigators in the field have devoted to verifying the pathogenesis. For instance, Gruszka et al. reported the hyperactivation of Wnt signaling cascades (e.g., β-catenin, phosphorylated-GSK3β) in haematopoietic stem/progenitor cells (HSPCs) in AML patients, and highlighted the necessity for the maintenance of leukemic stem cells (LSCs) and the prognostic value for tumor eradication [32]. Nilsson et al. verified the worse prognosis of therapy-related AML (t-AML) with mutated NPM1 over the relative subsets in cytogenetically intermediate- and adverse-risk [33]. Instead, our group illuminated the involvement of transcriptomic alterations and cellular vitality of bone marrow-derived mesenchymal stem/stromal cells (BM-MSCs), and in particular, the hyperactivation of JAK-STAT signaling pathway in the pathogenesis of AML [22]. In this study, we further put forward the potential pathogenicity of AML-NKs with multidimensional alterations in the cellular and omics characteristics, especially the sharp decline in the content of total NK cells (CD3^−^CD56^+^) and the activated CD25^+^ NK cell subset. In consistence, in this study, we also observed the variations in the aforementioned activated subsets of NK cells, together with the specific enrichment of gene sets involved in JAK-STAT cascade and inflammatory response (e.g., Treg, Th17, IL-6, IFN-α) in HD-NKs and AML-NKs, which collectively indicated the pathogenicity of JAK-STAT hyperactivation in AML. However, the systemic and detailed information of JAK-STAT signaling cascades in mediating AML-NK-related immunodysfunction and alterations in cellular viability (e.g., cell cycle, cell apoptosis) still need to be further illuminated. For instance, Wang and the colleagues reported the bone marrow NK (BM-NK) cell dysfunction by GARP-mediated TGF-β activation in AML patients, which indicated the pathogenicity of BM-NK cell-associated immunodysfunction in AML [34]. Interestingly, Haroun-Izquierdo et al. and Dong et al. respectively reported the adaptive single-KIR^+^NKG2C^+^ NK cells and CAR-transduced memory-like NK cells with potent missing-self reactivity upon HLA-mismatched AML and potent activity against NMP1 mutated AML, which collectively highlighted the feasibility of AML treatment by modulating specific NK cell subsets [35, 36]. Additionally, Paczulla et al. observed LSCs with absence of NKG2D ligands revealed immune evasion in AML, whereas Romee et al. and Parihar verified the therapeutic effect of memory NK cells for relapsed AML [37–39]. Therewith, it’s of interesting to further explore the biofunction and omics signatures of the aforementioned NK cell subsets for developing novel NK cell- and CAR-NK cell-based immunotherapy.

With the aid of transcriptomic analysis, we further observed that the DEGs between AML-NKs and HD-NKs were involved in immunoregulatory processes (e.g., IL-6 biosynthesis, immune response, antigen processing and presentation, graft-versus-host disease, inflammatory response, interferon response) and signaling cascades (e.g., IL6-JAK-STAT, KRAS signaling). Very recently, D'Silva et al. reviewed the implication of NK cell defects in AML progression and discussed the disease-associated mechanisms, and in particular the expression profiling of different cell surface markers of AML-NK cells [40]. The further exploration of the alterations in AML-NK cells would benefit our understanding upon pathogenesis and therapeutic regimens of AML from the aspect of numerical, receptor expression, and maturation defects of NK cells, along with checkpoint overexpression inhibitors and epigenetic modifications [40]. Of note, it’s of great interesting to further explore the potential variations of NK cells in bone marrow environment of AML patients before treatment and remission, which will help illuminate the biofunction and omics features of AML and benefit the development of targeted therapy in clinical practice.

State-of-the-art literatures have highlighted the involvement of dysimmunity in the pathogenesis and drug resistance of AML patients. For instance, Zhang et al. verified the multiple chemoresistant properties in LSCs and the oxidative phosphorylation (OXPHOS) signatures in pediatric AML [41]. Very recently, Naldini et al. reported the longitudinal single-cell profiling and functional validation of chemotherapy response in AML patients, and identified the variations in the stemness and quiescence signatures of the OxPhos^low^ miR-126^high^ LSCs [42]. Of note, Abbas et al. outlined the T cell receptor repertoire profiling and the T cell landscape in AML patients with PD-1 blockade therapy [43]. Instead, we conducted systematic and detailed characterization of the multifaceted signatures of NK cells and the variations in subsets and gene expression pattern between resident and expanded AML-NKs and HD-NKs. To our knowledge, there were minimal reports upon the specific subsets (e.g., CD3^−^CD56^+^CD25^+^) of AML-NKs in AML patients. Similarly, Stringaris and the colleagues verified the upregulation of NKG2A (a inhibitory receptor) and the downregulation of NKp46 in NK cells of AML patients, which indicated the long-lasting changes and effector function impairment in AML-NK cells induced by AML blasts [44]. Interestingly, Crinier and the colleagues revealed the trajectories and the stress signature of NK cell differentiation in bone marrow, which further indicated the involvement of AML-NKs in the pathogenesis of AML [45]. Therefore, it is of great interesting to further dissect the single-cell mutation and gene expression profiling of clonal evolution as well as cellular hierarchy in AML patients, which will collectively benefit the development of accurate diagnosis and targeted therapy [46, 47].

## Conclusion

Overall, in this study, we systematically and meticulously dissected the biological phenotypes, cellular vitality and cytotoxicity of both resident and expanded NK cells between AML-NKs and HD-NKs from the landscape of gene expression pattern and somatic variations. Our findings put forward the potential pathogenic role of AML-NK cells in bone marrow microenvironment (BME) in AML patients, which could be conducive to the further exploration of the pathogenesis of AML and the development of NK cell-based targeted therapy in future.

### Supplementary Information

**Additional file 1: Table S1.** The detail data of AML. **Table S2.** The cytokines used in this study. **Table S3.** Antibodies for flow cytometry assay in the study.

**Additional file 2: Figure S1.** Representative FCS raw data files for rHD-NKs and rAML-NKs.

**Additional file 3: Figure S2.** Antibody matching tables and representative FCS Raw Data files for cell apoptosis analysis of eHD-NKs and eAML-NKs.

## Data Availability

All data of this study are included in the published article. Meanwhile, the datasets analyzed during this study are available from corresponding author upon reasonable request.

## Abbreviations

BM-NKs: Natural killer cells from bone marrow
MNCs: Mononuclear cells
GvHD: Graft-versus-host disease
HSC: Hematopoietic stem cell
PBS: Phosphate buffer solution
PCA: Principal component analysis
GSEA: Gene set enrichment analysis
KEGG: Kyoto encyclopedia of genes and genomes
EMT: Epithelial-mesenchymal transition
NKCE: NK cell engager
ADCC: Antibody-dependent cytotoxicity
BME: Bone marrow microenvironment
VSEs: Variable shear events
DEGs: Differentially expressed genes
OXPHOS: Oxidative phosphorylation
BM-MSCs: Bone marrow-derived mesenchymal stem/stromal cells
t-AML: Therapy-related AML
LSCs: Leukemic stem cells
HSPCs: Haematopoietic stem/progenitor cells
ILCs: Innate lymphoid cells
