# Supplementary material for: Uncovering the cellular and omics characteristics of natural killer cells in the bone marrow microenvironment of patients with acute myeloid leukemia
Source: Cancer Cell Int. 2024 Mar 14;24:106. doi: 10.1186/s12935-024-03300-w (PMC10938822; doi:10.1186/s12935-024-03300-w)
Supplement: Supplementary file 1 — Additional file 1: Table S1. The detail data of AML. Table S2. The cytokines used in this study. Table S3. Antibodies for flow cytometry assay in the study. [file 12935_2024_3300_MOESM1_ESM.docx]

**Additional file**

**Uncovering the Cellular and Omics Characteristics of Natural Killer Cells in the Bone Marrow Microenvironment of Patients with Acute Myeloid Leukemia**

Leisheng Zhang^1,2^**^§^**, Yunyan Sun^3^**^§^**, Chun-e Xue^4^**^§^**, Shuling Wang^5^, Xianghong Xu^3^, Chengyun Zheng^6^, Cunrong Chen^5*^, Dexiao Kong^6*^

**Additional file**

**Table S1-S3**

**Legends for Figure S1-S2**

**Table S1. The detail data of AML**

| Grouping | Number | Gender | Age (year-old) |
| --- | --- | --- | --- |
| HD group | HD-1 | Male | 47 |
|  | HD-2 | Female | 53 |
|  | HD-3 | Female | 17 |
|  | HD-4 | Female | 27 |
|  | HD-5 | Male | 25 |
|  | HD-6 | Female | 49 |
|  | HD-7 | Female | 55 |
|  | HD-8 | Male | 50 |
|  | HD-9 | Female | 30 |
|  | HD-10 | Male | 28 |
| AML | AML-1 | Female | 54 |
|  | AML-2 | Female | 34 |
|  | AML-3 | Male | 20 |
|  | AML-4 | Male | 54 |
|  | AML-5 | Male | 46 |
|  | AML-6 | Male | 15 |
|  | AML-7 | Female | 53 |
|  | AML-8 | Female | 60 |
|  | AML-9 | Male | 22 |

**Table S2. The cytokines used in this study.**

| **Reagent** | **Cat. No.** | **Conc.** | **Source** |
| --- | --- | --- | --- |
| Recombinant Human IL-2 (rhIL-2) | 200-02 | 100 ng/uL | PeproTech Inc, USA |
| Recombinant Human IL-15 (rhIL-15) | 200-15 | 10 ng/uL | PeproTech Inc, USA |
| Recombinant Human IL-18 (rhIL-18) | 119-BP-100 | 10 ng/uL | R&D Systems, USA |

**Table S3. Antibodies for flow cytometry assay in the study.**

| **Antibody** | **Cat. No.** | **Source** |
| --- | --- | --- |
| Anti-CD3-PE | 981004 | BioLegend |
| anti-CD3-APC | 317318 | BioLegend |
| Anti-CD25-FITC | 356106 | BioLegend |
| Anti-CD4-PE | 357403 | BioLegend |
| Anti-CD8-PE-Cy7 | 344711 | BioLegend |
| Anti-CD16-FITC | 302005 | BioLegend |
| Anti-NKG2D-perCP-Cy5.5 | 320817 | BioLegend |
| Anti-CD56-APC | 362503 | BioLegend |
| Anti-CD56-perCP-Cy5.5 | 362505 | BioLegend |
| Anti-CD107a-PE-Cy7 | 328617 | BioLegend |
| 7-AAD | 559925 | BD Pharmigen |
| PE anti-human IgG | 409304 | BioLegend |
| Percision Count Beads | 424902 | BioLegend |
| DAPI | MBD0015 | Sigma-Aldrich |
| Cell Cycle and Apoptosis Detection Kit | C1052 | Beyotime Biotehnology |
| CellTrace Voilet | C34557 | Invitrogen™ |
| Annexin V-FITC | AO2001-02G | Tianjin Sungene Biotech |
| Annexin V binding buffer (10X) | AB2000-G | Tianjin Sungene Biotech |
| FITC Annexin V-PI kit | 640914 | Biolegend |
| Anti-CD25-APC | 302609 | Biolegend |
| PE-Foxp3 | 12-5773-82 | eBioscience |
| anti-CD3-FITC | 317305 | Biolegend |
| Anti-CD4-APC | 300514 | Biolegend |
| APC-Cy7-FVD | L34971 | Invitrogen |
| Anti-CD4-APC-Cy7 | 344615 | Biolegend |
| Anti-NKp44-APC-Cy7 | 325123 | Biolegend |
| Anti-NKp46-PE-Cy7 | 331915 | Biolegend |

**Supplementary Figure Legends for Figure S1-S2**

**Figure S1.** Representative FCS raw data files for rHD-NKs and rAML-NKs.

**A-B.** Representative FCS raw data for total CD3^-^CD56^+^ NK cells and the indicated subsets of activated NK cells in rHD-NKs and rAML-NKs, including CD3^-^CD56^+^NKp44^+^ (**A**) and CD3^-^CD56^+^NKp44^+^ (**B**). **C.** Representative FCS raw data for the indicated T cell subsets, including CD4^+^ T cells, CD8^+^ T cells, and Treg.

**Figure S2.** Antibody matching tables and representative FCS Raw Data files for cell apoptosis analysis of eHD-NKs and eAML-NKs.

**A.** Antibody matching tables for the detection of the indicated subsets in eHD-NKs and eAML-NKs. **B.** Representative FCS raw data for cell apoptosis analysis in CD3^-^CD56^+^ eHD-NKs and eAML-NKs.
