# Supplementary figures and images for: Uncovering the cellular and omics characteristics of natural killer cells in the bone marrow microenvironment of patients with acute myeloid leukemia
Source: Cancer Cell Int. 2024 Mar 14;24:106. doi: 10.1186/s12935-024-03300-w (PMC10938822; doi:10.1186/s12935-024-03300-w)

A

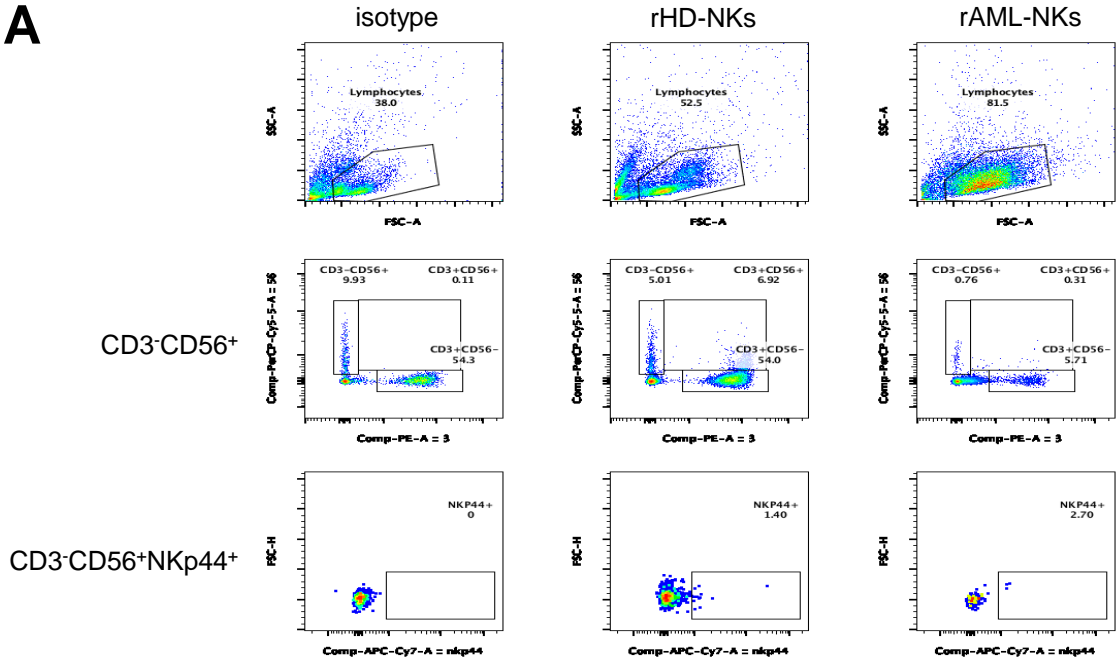

B

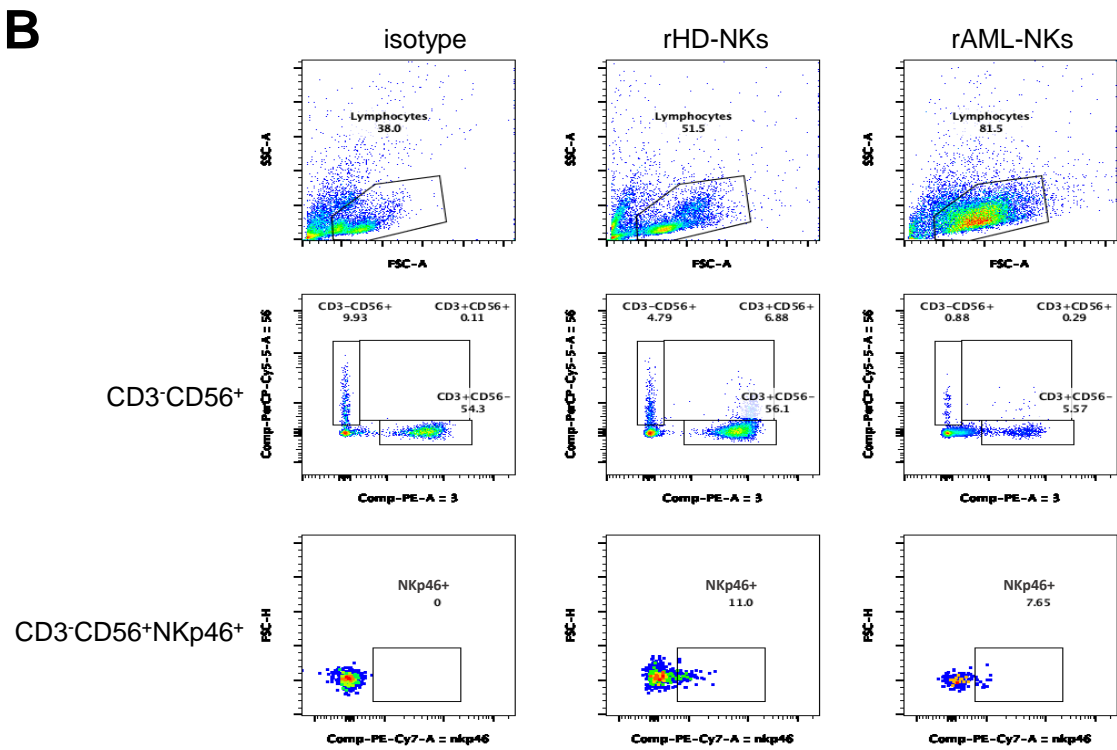

C

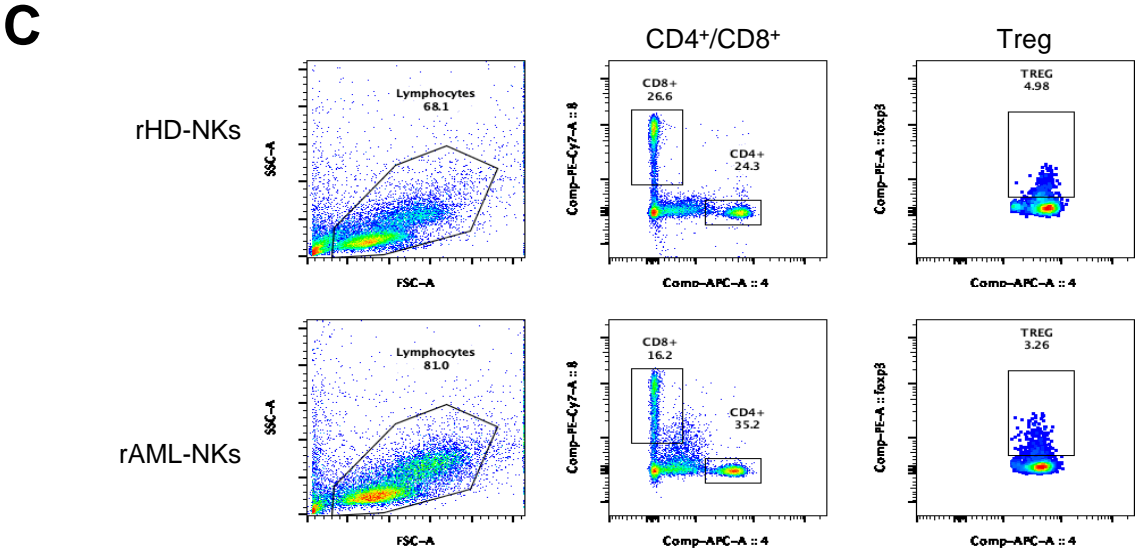

Supplement: Supplementary file 2 — Additional file 2: Figure S1. Representative FCS raw data files for rHD-NKs and rAML-NKs. [file 12935_2024_3300_MOESM2_ESM.pdf]
