# Supplementary material for: Uncovering the cellular and omics characteristics of natural killer cells in the bone marrow microenvironment of patients with acute myeloid leukemia
Source: Cancer Cell Int. 2024 Mar 14;24:106. doi: 10.1186/s12935-024-03300-w (PMC10938822; doi:10.1186/s12935-024-03300-w)

A

Antibody matching scheme 1
CD3-PE
CD56-APC
CD16-FITC
CD314 (NKG2D)-perCP-Cy5.5
CD4-APC-Cy7
CD8-PE-Cy7

Antibody matching scheme 3
foxp3-PE
CD56-perCP-Cy5.5
CD3-FITC
CD4-APC
CD8-PE-Cy7
FVD-APC-Cy7

Antibody matching scheme 2
CD3-PE
CD56-perCP-Cy5.5
NKp44-APC-Cy7
NKp46-PE-Cy7
CD25-APC

Antibody matching scheme 4
CD3-PE
CD56-APC
Annexin V-FITC
7AAD-perCP-Cy5.5

B

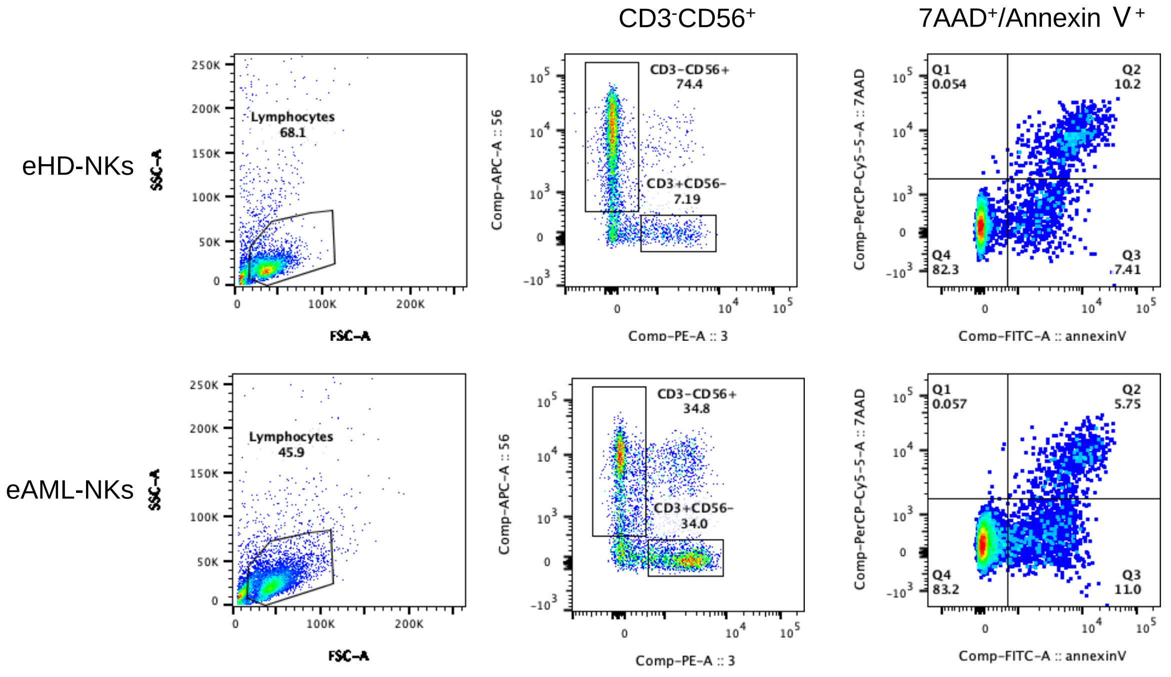

Supplement: Supplementary file 3 — Additional file 3: Figure S2. Antibody matching tables and representative FCS Raw Data files for cell apoptosis analysis of eHD-NKs and eAML-NKs. [file 12935_2024_3300_MOESM3_ESM.pdf]
